# Microstructural network alterations of olfactory dysfunction in newly diagnosed Parkinson’s disease

**DOI:** 10.1038/s41598-017-12947-7

**Published:** 2017-10-02

**Authors:** Ming-Ching Wen, Zheyu Xu, Zhonghao Lu, Ling Ling Chan, Eng King Tan, Louis C. S. Tan

**Affiliations:** 1 Department of Research, National Neuroscience Institute, Singapore, Singapore; 2 Department of Neurology, National Neuroscience Institute, Singapore, Singapore; 3 Department of Diagnostic Radiology, Singapore General Hospital, Singapore, Singapore; 4 Duke-NUS Medical School, Singapore, Singapore

## Abstract

Olfactory dysfunction is a robust and early sign for Parkinson’s disease (PD). Previous studies have revealed its association with dementia and related neural changes in PD. Yet, how olfactory dysfunction affects white matter (WM) microstructure in newly diagnosed and untreated PD remains unclear. Here we comprehensively examined WM features using unbiased whole-brain analyses. 88 newly diagnosed PD patients without dementia (70 with hyposmia and 18 without hyposmia) and 33 healthy controls underwent clinical assessment and diffusion tensor imaging (DTI) scanning. Tract-based special statistics (TBSS), graph-theoretic methods and network-based statistics (NBS) were used to compare regional and network-related WM features between groups. TBSS analysis did not show any differences in fractional anisotropy and mean diffusivity between groups. Compared with controls, PD patients without hyposmia showed a significant decrease in global efficiency, whilst PD patients with hyposmia exhibited significantly reduced global and local efficiency and additionally a disrupted connection between the right medial orbitofrontal cortex and left rectus and had poorer frontal-related cognitive functioning. These results demonstrate that hyposmia-related WM changes in early PD only occur at the network level. The confined disconnectivity between the bilateral olfactory circuitry may serve as a biomarker for olfactory dysfunction in early PD.

## Introduction

Mounting evidence has suggested that olfactory dysfunction is an early and consistent non-motor symptom in Parkinson’s disease (PD), affecting 45~90% of PD patients^1^. Diminished olfactory function may precede the onset of the classic motor symptoms in PD^2–4^, thereby being considered as a useful marker for early detection of PD. Patients with PD and hyposmia (hypos-PD) are prone to cognitive impairment^5,6^, cardiovascular dysautonomia^7,8^, and even dementia^9^. Understanding the neural substrates of olfactory dysfunction in PD is therefore of great clinical significance.

Previous single photon emission computed tomography (SPECT) or positron emission tomography (PET) studies have shown that olfactory dysfunction in PD is associated with altered metabolic activities in the limbic (e.g., the amygdala and hippocampus), occipital, and striatal areas^2,5,9–13^. In addition, functional MRI studies also indicate the presence of altered neural activity underlying olfactory dysfunction in PD. Specifically, patients with hypos-PD showed reduced activation in the bilateral rectus, orbitofrontal cortex (OFC), amygdala and parahippocampus and reduced functional connectivities between the left rectus and prefrontal, temporal, occipital, and limbic areas, compared with healthy controls (HCs)^14^. Structural MRI studies using region-of-interest (ROI) methods have unraveled the neuroanatomical basis of the metabolic and functional changes and have found that olfactory-related atrophy occurred not only in the primary olfactory cortex (e.g., the piriform and amygdala), but also extended to the OFC, temporal, parietal-occipital, and cingulate cortex in PD^9,15–17^.

Few studies have addressed the white matter (WM) correlates of olfactory dysfunction in PD. The work by Ibarretxe-Bilbao *et al*. employing diffusion tensor imaging (DTI) techniques to examine some pre-defined ROIs and discovered diminished WM integrity in the rectus and primary olfactory cortex of PD patients with severe olfactory dysfunction, when compared with PD patients without hyposmia (norms-PD) and HCs^18^. Olfactory performance was also found to be associated with WM features of the cerebellum^19^, but not the substantia nigra^20^. Most previous DTI studies on PD-related hyposmia examined only a few pre-selected brain regions^18,20,21^ or did not differentiate between hyposmic and normosic patients^19–23^. To date, there are no known studies examining the whole-brain WM network alterations that underlie olfactory dysfunction in PD, especially in early PD. The human brain can be conceptualised as an integrated large-scale network. The current focus in neuroimaging research is on the study of integrated models of brain structure and function, rather than a set of independently operating brain areas^24^. Graph-theoretical approaches allow one to characterise the segregation and integration within the nodes (i.e., brain regions) of the structural network^25^. Network-based statistics additionally offer complementary information about specific features within a network that can be applied to elucidating the structural and functional organisation of the brain^26^.

In the current study, we used DTI to investigate the whole brain to determine whether olfactory dysfunction would compromise WM regional and network properties in early PD. We hypothesised that WM would be altered at the network level, but not regional level, in early PD patients with olfactory dysfunction.

## Results

### Demographic and clinical findings

There were no significant group differences in performance on all cognitive tests, except the Symbol-Digit Modalities Test (SDMT, p < 0.05). Post-hoc analysis showed that hypos-PD patients performed worse than HCs on this test, but no difference was found between the two PD groups and between norms-PD and HC groups. As expected, a significant group difference was found in olfactory function (p < 0.001). Post-hoc analysis indicated that both HCs and norms-PD patients had higher University of Pennsylvania Smell Identification Test (UPSIT) scores than hypos-PD patients, whilst no significant difference existed between the former two groups. In addition, no significant differences were noted in disease duration, Hoehn & Yahr (H&Y) staging, and motor severity between the two PD groups. Detailed demographic and clinical data and statistics are presented in Table 1.

**Table 1:** Demographic and clinical characteristics.

	HC (n = 33)	Norms-PD (n = 18)	Hypos-PD (n = 70)	P value
	*Mean (SD)*	*Mean (SD)*	*Mean (SD)*	
Age (years)	57.93 (11.30)	57.66 (9.26)	59.69 (7.84)	0.54
Gender, male (%)^†^	60.6	38.9	61.4	0.21
Education (years)	15.70 (3.18)	15.72 (2.52)	15.40 (3.08)	0.86
Handedness, N (R/L/M)^†^	25/ 7/1	16/2/0	65/3/2	0.072
PD duration (months)^‡^	—	7.44 (7.53)	6.79 (7.15)	0.73
H&Y, N (1/2)^†^	—	9/ 9	24/ 46	0.17
UPDRS-III	—	20.06 (8.30)	20.71 (9.15)	0.78
UPSIT^*^	36.39 (1.60)	35.94 (1.39)	17.53 (5.85)	<0.001
MOCA	28.33 (1.19)	27.50 (2.38)	27.63 (2.33)	0.23
GDS	1.33 (1.78)	2.22 (2.51)	2.47 (2.55)	0.08
HVLT-Immediate Recall	47.39 (10.51)	53.39 (9.36)	48.61 (13.08)	0.22
HVLT-Delayed Recall	48.88 (10.22)	49.28 (7.80)	50.36 (12.85)	0.82
HVLT-Recognition	50.60 (8.82)	50.00 (9.07)	50.80 (10.77)	0.96
Benton JOL	13.00 (2.41)	12.53 (2.18)	12.00 (2.99)	0.22
LNS	11.79 (2.41)	12.50 (1.76)	11.86 (2.78)	0.61
SF (Animal)	54.00 (9.46)	53.00 (7.99)	51.67 (9.71)	0.49
SDMT^§^	51.74 (12.58)	46.43 (7.64)	45.95 (9.32)	0.03
Head motion				
Translation (mm)	0.43 (1.90)	0.53 (0.25)	0.45 (0.19)	0.2
Rotation (degree)	3.06 × 10^−3^ (2.47 × 10^−3^)	3.70 × 10^−3^ (3.13 × 10^−3^)	3.21 × 10^−3^ (1.93 × 10^−3^)	0.62
Absolute motion (mm)	1.53 (0.31)	1.66 (0.34)	1.55 (0.25)	0.27

Note: all analyses were one-way ANOVA, except ^†^using *χ*² or Fisher’s Exact test and ^‡^using independent-samples t test. *Post-hoc analysis indicated significant differences between HC and hypos-PD groups and between norms-PD and hypos-PD groups, but no difference between HC and norms-PD groups. ^§^Post-hoc analysis indicated significant differences between HC and hypos-PD groups; GDS = Geriatric Depression Scale; Handedness: R = right, L = left, M = Mixed; HVLT = Hopkins Verbal Learning Test; H&Y = Hoehn & Yahr staging; JOL = Judgment of Line Test; LNS = Letter-Number Sequencing Test; MOCA = Montreal Cognitive Assessment; SDMT = Symbol-Digit Modalities Test; SF = Semantic Fluency Test; UPDRS-III = MDS-Unified Parkinson’s Disease Rating Scale-Motor Subscale; UPSIT = University of Pennsylvania Smell Identification Test.

### TBSS analysis

There were no significant group differences in head motion (ps > 0.05, Table 1). We did not find any regions showing significant group differences in fractional anisotropy (FA) and mean diffusivity (MD) (ps > 0.05).

### Graph-theoretical analysis

**Figure 1:**
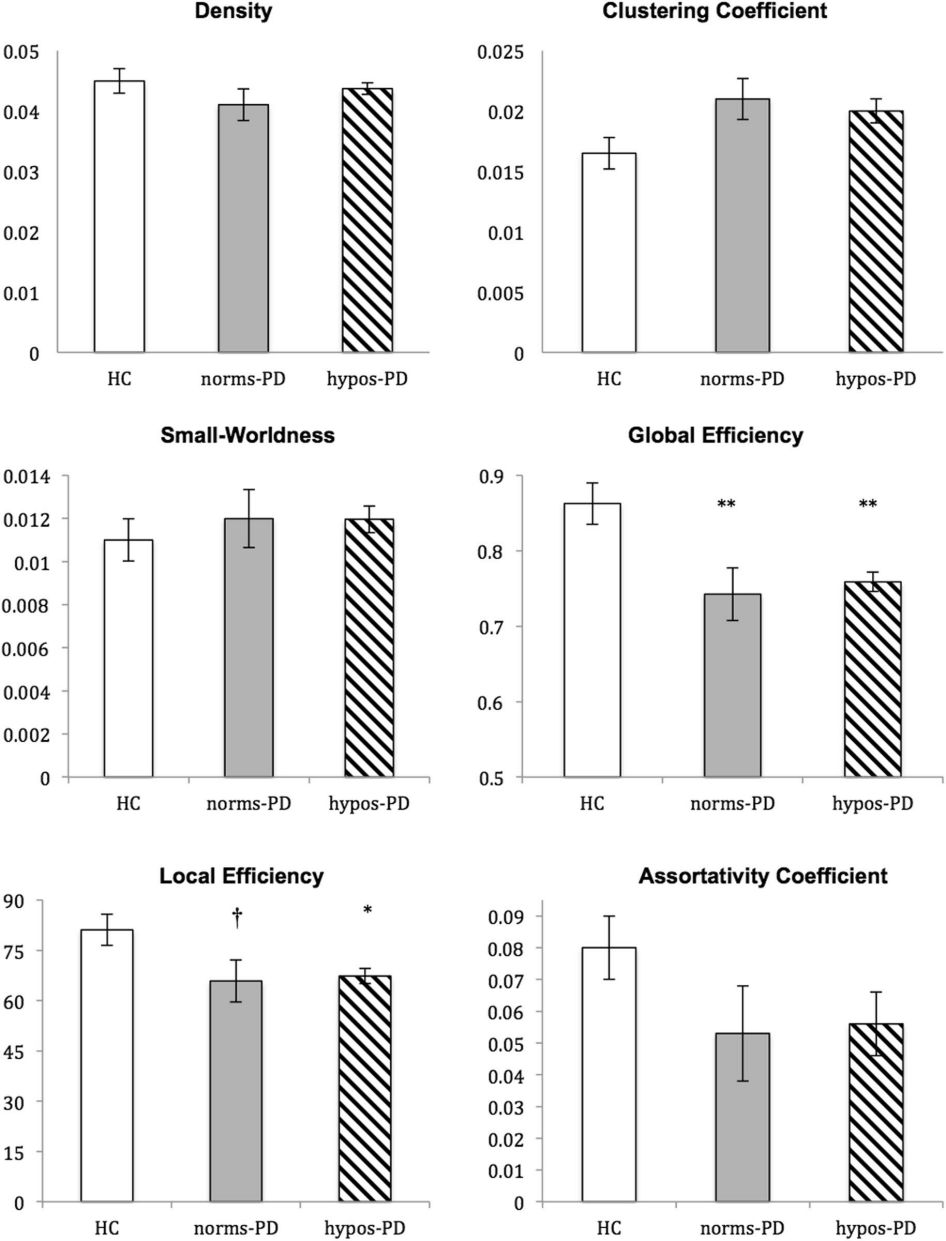
Group averages and comparisons of topological properties (Note: *p < 0.05 compared with HC, **p < 0.01 compared with HC, ^†^0.05 < p < 0.10 compared with HC; norms-PD = PD patients without hyposmia; hypos-PD = PD patients with hyposmia).

The overall topology of the networks showed that significant group differences were found to be in global efficiency (p = 0.001) and local efficiency (p = 0.026). Post-hoc analyses showed that compared with the HC group, the hypos-PD group had significantly decreased global efficiency and local efficiency (p < 0.01 and < 0.05, respectively), and the norms-PD group exhibited significantly reduced global efficiency (p < 0.01) but only slightly reduced local efficiency (p = 0.052). No significant differences were found between the two patient groups and in other graph theoretical metrics. Figure 1 presents the box plots of group averages of the graph-theoretic indices.

### NBS of structural connectivity

**Figure 2:**
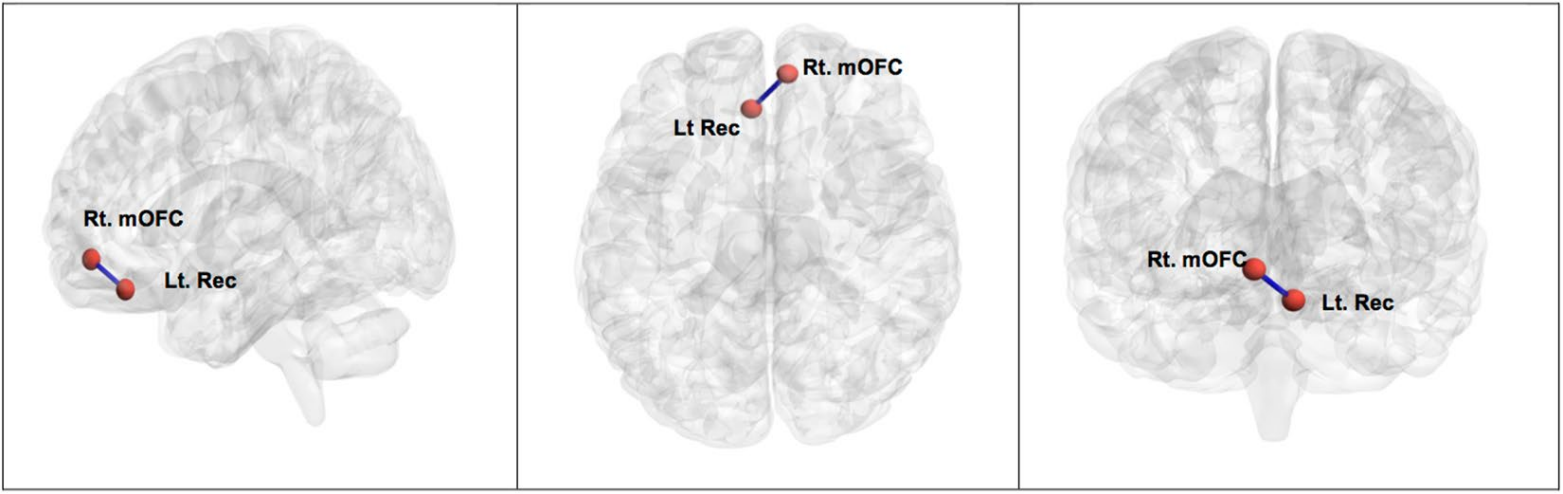
Subnetwork between the right medial orbitofrontal cortex (Rt. mOFC) and left rectus (Lt Rec) where PD patients with hyposmia (hypos-PDs) showed decreased connectivity, compared with healthy controls (HCs).

NBS analysis revealed a single network of decreased structural connectivity in the hypos-PD group as compared with the HC group (t = 3.662, p < 0.01). The network comprised a total of 2 nodes, including the right medial orbitofrontal cortex (mOFC) and the left rectus (Fig. 2). In contrast, the norms-PD group did not show any alterations of structural connectivity when compared with HC and hypos-PD groups.

### ROC curve analysis

Global efficiency and local efficiency values enabled statistically significant differentiation between HCs and norms-PDs (area under the ROC curve [AUC] was 0.74 and 0.67, respectively; p = 0.006 and 0.042, respectively; overall sensitivity = 70% and specificity = 67%). In addition, global efficiency, local efficiency, and connectivity between the right mOFC and left rectus demonstrated good AUC values in distinguishing HCs and hypos-PDs (AUC = 0.75, 0.71, and 0.64, respectively; p < 0.001, = 0.001 and = 0.03, respectively; overall sensitivity = 67% and specificity = 74%). In contrast, there was no significant classifier for differentiating the two PD groups.

### Correlation analysis

The correlation analysis revealed that global efficiency, local efficiency, and connectivity between the right mOFC and left rectus did not significantly correlate with UPSIT or SDMT in the two patient groups.

## Discussion

In this study, we comprehensively examined the whole brain for hyposmia-associated WM changes in newly diagnosed PDs using TBSS, graph-theoretical and network-based analyses. Findings from our study revealed no significant regional changes as reflected by FA and MD but observable alterations at the network level as evidenced by reduced global and local efficiency in the early disease stages of PD. Hypos-PD patients additionally showed reduced connectivity within a single network that comprised the right mOFC and left rectus, compared with HCs.

Prior studies have observed FA reduction in the rectus^18^ and reduced functional activation and connectivity in the OFC and rectus regions in hypos-PD patients^14^. In this study, we demonstrated that structural connection between these two regions was disrupted in early hypos-PD patients, thereby providing further support to the literature. Reduced connectivity strength between the right mOFC and left rectus also showed significant power to differentiate hypos-PD patients from HCs. Unlike previous functional MRI studies that showed widespread alterations of functional connectivity in PD patients with hyposmia^14,27,28^, we only observed a single microstructural network breakdown in our DT study. The explanations could be first, the use of different imaging modalities and different disease stages of patients in our study and in other studies. According to a recent report, subtle structural network alterations but more prominent functional network reduction were found in early PD^29^. Also, findings from prior neuroimaging studies in olfactory dysfunction in PD (see Supplementary Table S1) indicated that in general, studies using functional imaging, including PET and SPECT, observed more significant brain areas relating to olfactory impairment, compared with structural imaging studies. Therefore, it would be reasonable to expect less salient change in WM structural networks in early PD patients with hyposmia. Second, different imaging analytical approaches may also account for discrepant findings. For instance, ROI-based studies only examined few pre-selected brain regions and hence usually do not apply correction methods for multiple comparisons. In contrast, studies examining the whole brain are deemed to correction for multiple comparisons. Different analytical approaches and correction methods for multiple comparisons have different operations to determine statistical significance, which is likely to lead to different results. Nonetheless, hyposmia-related brain regions found in our current study on early and *de novo* PD were in good agreement with previous observations^14,18,19,30,31^.

Similarly, the fact that we did not find any group difference in regional WM characteristics or substantial changes in some graph-theoretical metrics, such as density and small-worldness, could be accounted for by the early disease stages of our patient participants who may have subtle structural changes. This view is supported by a recent DTI study that found limited changes in the global network metrics of early PD patients^32^. Although structural and functional connections in cellular networks may undergo dynamic changes over the span of human development, ageing, or disease progression, cellular functional networks are more responsive to transient synchronisation that occur within a short period of time^33^. As such, the view that relatively slow structural modifications are accompanied by faster changes in functional connections seems plausible. Notably, although there were some structural changes in early PD, especially among those with hyposmia, the changes were confined to the network level, suggesting that hyposmia-associated WM changes in early disease stages may be primarily related to network disconnection, but did not occur at the regional level.

Large-scale neural networks in the small-world model are geared towards a high global and local efficiency to minimise the consumption of energy and resources required for effective information processing^24^. Global efficiency is considered a good measure of the overall capacity for parallel information transfer and integrated processing^34^. Local efficiency quantifies the resistance of a network to failure on a small scale^24^. Reduced efficiency at both global and local levels was found in early PD and was able to differentiate PD patients from HCs, thus suggesting that compromised neural information processing and neural resistance to local damage exited in early PD.

Consistent with previous literature, the majority of newly diagnosed PD patients in our study suffered olfactory dysfunction, thereby supporting the view that olfactory dysfunction is a consistent sign in early PD^2–4^. Despite being without significant cognitive impairment, hypos-PD patients showed poorer performance on the SDMT, compared with matched HCs. The SDMT is a measure of attention, visual scanning, and cognitive speed^35^. Successful performance on this test would require the frontal-related regions to be engaged^36^. This association between frontal-related cognitive dysfunction and olfactory dysfunction in PD have been reported previously^6^. As we found that hypos-PD patients had disrupted connection between the bilateral frontal regions, the impaired neuropsychological profile of this patient group was therefore in good agreement with the altered network features. As mild cognitive impairment (MCI) in PD involves frontostriatal dysfunction^37^, the observation of frontal cognitive impairment in the hypo-PD group implies that these patients may have a higher risk of developing MCI. Although the association between hyposmia and MCI due to Alzheimer pathology has been identified^38^, it remains understudied in PD. The cholinergic system is known to be associated with cognition in Alzheimer’s disease (AD) and PD, according to the work by Xu *et al*.^39^. Two recent studies revealed that PD patients with olfactory dysfunction exhibited cholinergic system degeneration, compared with norms-PD patients and HCs^40,41^. Further, cholinergic integrity in the limbic areas (e.g., hippocampal formation and amygdala) and neocortex was associated with olfactory performance in PD^5^. These pieces of evidence may explain why we found worse cognitive function in early PD patients with olfactory impairment. Longitudinal studies would be needed to clarify the specific associations of olfactory dysfunction with different subtypes of MCI in PD.

The underlying mechanisms of hyposmia in PD may be manifold. Previous works have indicated the relationship between cardiovascular dysautonomia and olfactory dysfunction in PD^7,8^. Such a relationship was not found in atypical Parkinsonian diseases (e.g., multiple system atrophy)^42^, thereby suggesting the exclusive role of autonomic network failure in PD-related hyposmia. In addition, neuroinflammation in the olfactory bulb might promote the aggregation of α-synuclein and thus lead to PD neuropathology^43^. Hyposmia may precede motor symptoms and is associated with dopaminergic abnormalities in the olfactory bulb in prodromal PD^44^. As anti-inflammatory therapies could slow disease progression^43^, future studies should examine the impact of neuroinflammation on hyposmia in the prodromal phase of PD and determine whether the use of anti-inflammatory medications in the prodromal phase may prevent the development of PD.

The strengths of our study were as follows. First, all PD patients in the study underwent DAT imaging to confirm the standard clinical diagnosis of PD. Second, we carefully controlled for head motion in DTI analysis and further ensured no group difference in head motion to reduce the confounding effects caused by motion. Finally, we adopted unbiased whole-brain analytical approaches to thoroughly examine WM properties of hypos-PD patients. One limitation of our study was the lack of functional or perfusion imaging to determine whether the alterations of neural activity relating to olfactory dysfunction would be more marked than structural network changes in this early PD cohort. A recent study demonstrated that the combination of DTI and arterial spin labeling modalities enabled higher accuracy of differentiating early PD patients from HCs^45^. It would be interesting to investigate whether such a combination will identify more sensitive and specific brain markers of hyposmia in PD. Although there have been few studies using functional MRI to examine the neural functional substrates of olfactory dysfunction in PD^14,27,28^, no known studies have examined the hyposmia-associated functional network properties in PD. Second, hyposmia is found not only in PD, but also in other neurological disorders, such as AD^46,47^. As we did not have other patient populations who also suffer hyposmia in the study, we were not able to compare the properties of the bilateral olfactory circuitry of different neurodegenerative patients with hyposmia, which was another limitation of the study. Finally, findings from our current work were based on cross-sectional observations and hence have prevented us from making any inference about the trajectory of WM network alterations in hypos-PD patients, and the impact of these changes on cognition as the disease progresses.

## Conclusions

We show for the first time that in addition to the breakdown of the efficiency of information transfer and resistance to local neural deficits, a subnetwork connecting the right mOFC and left rectus was disrupted, whereas no regional changes were observed in early hypos-PD patients. By examining whole-brain regional and network topology of early PD patients, our findings suggest that compromised connectivity of the right mOFC and left rectus uniquely differentiates hypos-PD patients from HCs, and thus serve as a potential biomarker for hyposmia in early PD. Future studies may expand on this work by assessing whether alterations of this subnetwork can predict cognitive decline in hypos-PD patients.

## Materials and Methods

### Participants

The data of all participants, including newly diagnosed PD patients and HCs, in the current study were obtained from the Parkinson’s Progression Markers Initiative (PPMI, http://www.ppmi-info.org/). The PPMI is an observational, international, multi-center study designed to identify PD progression biomarkers. The study was approved by the Institutional Review Board of each participating site. Written informed consent was obtained from all participants before study enrollment. The study was performed in accordance with the relevant PPMI guidelines and regulations. Our manuscript was reviewed and approved by the PPMI Data and Publications Committee.

To be enrolled into the PPMI study, all patients were required to fulfill the following criteria: 1) met the standard diagnostic criteria for PD, 2) diagnosed within 2 years before the initial visit, 3) H&Y stage ≤ 2 at baseline, 4) demonstrated deficits of dopamine transporters (DATs) on single-photon emission computed tomography (SPECT) imaging, and 5) not on any PD medication at baseline. All healthy controls (HCs) were required to have normal DATs and be free of any significant neurological disorders and medications that might interfere with the results of DAT SPECT imaging. We only included demographically matched participants without dementia or psychiatric conditions but with quality DTI data in our analysis. Given the inclusion criteria, a total of 121 participants (18 norms-PDs, 70 hypos-PDs, and matched 33 HCs) with good quality DTI were included in the analysis. Compared with previous DTI studies in PD-related hyposmia^18–20,48,49^, the sample size in our study was the largest and therefore should provide sufficient statistical power for detecting group effects.

### Clinical assessments

Following initial screening, all participants were comprehensively assessed at the baseline visit for clinical performance on motor, non-motor, cognitive, and neuropsychiatric functions by the site investigators. General motor severity was evaluated using the Unified Parkinson’s Disease Rating Scale (UPDRS-III)^50^. Global cognitive function was assessed using the Montreal Cognitive Assessment (MOCA)^51^. Neuropsychiatric assessment was performed using the 15-item Geriatric Depression Scale (GDS)^52^. In addition, detailed cognitive functions were assessed using the Hopkins Verbal Learning Test^53^, Benton Judgment of Line Orientation Test^54^, Letter-Number Sequencing Test^55^, Semantic Fluency (Animal) Test, and Symbol-Digit Modalities Test (SDMT)^56^.

Olfactory identification was evaluated with the UPSIT^57^. The participants were presented with forty different odors and tasked to make a force choice from four possible answers. According to the norm in the UPSIT manual^58^, normal olfactory function is characterised by having a score of 33 and greater, whilst a score below 33 is considered hyposmia.

### Image acquisition

Image acquisition was performed using a standardised protocol on 3 T Siemens scanners (all Siemens Healthcare, Malvern, PA). Details of the MRI acquisition can be found in the MRI technical operations manual at http://www.ppmi-info.org/. In brief, a 2-dimensional echo-planar DTI sequence was acquired for each participant using the following parameters: TR/TE = 900/88 ms, flip angle = 90°, voxel size = 2 × 2 × 2 mm^3^, 72 slices, 64 gradient directions with a b-value of 1000 s/mm^2^. One non-gradient volume (b = 0 s/mm^2^) was also acquired. The MRI protocol was distributed to each recruiting site to ensure consistent installations.

### Image preprocessing

All DTI data was preprocessed using FSL 5.0.7 (http://fsl.fmrib.ox.ac.uk/fsl). Eddy currents and head motion were corrected by affine registration to the first b0 image using the ‘eddy_correct’ function in FSL. The movement of each participant in terms of x, y, and z coordinates was computed based on the output of eddy_correct. A brain mask was created using the fractional intensity threshold of 0.3 to ensure that only diffusion tensors inside the brain were computed. The diffusion tensors were then linearly fitted to the diffusion-weighted images using the ‘dtifit’ tool in FSL, generating maps of FA and MD.

In addition, a measure of volume-to-volume displacement was obtained for each participant using the output file of the ‘eddy_correct’ motion correction function. To reduce the effect of excessive head motion during DTI acquisition, only participants who showed limited head movement during imaging acquisition (i.e., translation: < 3 mm; rotation: < 2 degrees; maximum absolute head motion: < 4 mm) were included in the study.

### Tract-Based Spatial Statistics (TBSS) analysis

The TBSS tool in FSL^59^ was used to compare DTI measures between PD patients and HCs and to correlate significant DTI measures with motor dysfunction. First, all participants’ FA maps were nonlinearly aligned to the predefined FSL FMRIB58 FA map and registered to MNI152 standard space. The mean FA skeleton, a representation of the centre of the WM tracts common to all participants was created and thresholded at FA > 0.20. The aligned FA map of each participant was projected onto the FA skeleton. A similar process was done for the MD maps, such that without the initial registration, the MD maps were projected onto the mean FA skeleton.

### Network construction

A brain network can be described as a graph with nodes representing brain regions and edges that form connections between the nodes. The nodes were defined using the Automated Anatomical Labeling (AAL) atlas, which included a total of 116 cortical and subcortical regions. Motion-corrected DTI data was first reconstructed in the MNI space using q-space diffeomorphic reconstruction (QSDR) to obtain the spin distribution function. A diffusion sampling length ratio of 1.25 was used^60^. Whole-brain deterministic tractography was performed in DSI Studio (http://dsi-studio.labsolver.org). Fiber tracking was stopped if the reconstructed fiber entered a voxel with quantitative anisotropy (QA) of less than 0.2, and if the streamline made a turn with a curvature angle of more than 45 degrees. Tracts with length less than 30 mm were discarded. The overall pattern of WM connections between each pair of brain nodes was computed using weighted matrices. In the weighted matrices, each edge represented the product of the count of the connection tract. The connectivity matrices and graph theoretical analysis were conducted using the DSI Studio and Brain Connectivity Toolbox^25^. The following global network metrics were investigated: network density, clustering coefficient, small-worldness, global efficiency, local efficiency, and assortativity coefficient.

Network-based statistics (NBS)^26^ was used to further localise specific pairs of brain regions where WM structural connectivity was altered in hypos-PD patients. General linear models were used to examine the mean difference in connectivity strength (defined by QA) of any connected components between groups. A corrected p value was calculated for each component, using the null distribution of maximal connected component size derived with a nonparametric permutation method.

### Statistical analysis

Demographical and clinical data, and head motion during DTI acquisition were analysed using IBM Statistical Package for Social Sciences software (version 21; SPSS, Inc., Chicago, IL). For continuous variables, one-way analysis of variance (ANOVA) and independent-sample t tests were used to compare group differences where appropriate. Pearson’s chi-squared or Fisher’s exact tests were used to compare categorical variables.

A general lineal model using ‘randomise’ in FSL was performed to test group differences in FA and MD between the hypos-PD, norms-PD and HC groups, whilst controlling for UPDRS-III. This program used permutation-based testing with 5000 permutations and statistical inference by applying threshold-free cluster enhancement (TFCE)^61^ with a threshold of p < 0.05, corrected for multiple comparisons.

The comparisons of global network metrics between groups were examined with ANCOVA controlling for UPDRS-III. P < 0.05 was deemed to be significant. To determine the significance levels of altered connectivity networks in NBS analysis, we performed a general linear model controlling for UPDRS-III at each edge independently to test for group differences in connectivity. A threshold (p = 0.05) was used to form a set of suprathreshold edges among which any connected components and their size (number of edges) could be determined. The statistical significance of the size of each observed component was assessed with respect to an empirical null distribution of maximal component sizes obtained under the null hypothesis of random group membership (5,000 permutations). Significant subnetworks were determined at p < 0.05 (corrected).

Receiver operating characteristic (ROC) curve analysis with leave-one-out cross validation was used to quantify the ability of significant global network metrics and subnetwork connectivity to enable discrimination between groups with the significance level being set at p < 0.05.

Finally, to assess whether differences in olfactory and cognitive functions between PD patients with and without hyposmia might be associated with regional and network WM changes, correlation analyses between significant WM changes and UPSIT and significant cognitive variables were performed across the two patients groups using Pearson’s linear correlations (corrected for UPDRS-III, p < 0.05 as the significance threshold).

## Electronic supplementary material

Supplementary Information
