# Supplementary material for: Microstructural network alterations of olfactory dysfunction in newly diagnosed Parkinson’s disease
Source: Sci Rep. 2017 Oct 2;7:12559. doi: 10.1038/s41598-017-12947-7 (PMC5624890; doi:10.1038/s41598-017-12947-7)
Supplement: Supplementary file 1 — Supplementary Information [file 41598_2017_12947_MOESM1_ESM.pdf]

Microstructural network alterations of olfactory dysfunction in newly diagnosed Parkinson's disease

Ming-Ching Wen, Zheyu Xu, Zhonghao Lu, Ling Ling Chan, Eng King Tan, Louis CS Tan

Supplementary Table S1. Previous neuroimaging studies on olfactory dysfunction in PD

Study	Subjects	Modality	Analytical Method	Imaging Index	Key findings
Baba et al. (2012)[1]	20 SH-PDs<br>24 NH-PDs	PET, CT	WB	Metabolism, atrophy	• Baseline:<br>NH-PD: mild metabolic reduction in frontal, medial occipital cortices, but no cortical atrophy<br>SH-PD: ↓ metabolism in bil. DLPFC, medial prefrontal & occipital cortices, parieto-occipital-temporal areas and ↑ atrophy in bil. prefrontal, medial prefrontal, temporal, cingulate, occipital cortices and precuneus<br>• 3-year follow-up:<br>NH-PD: ↓ metabolism in midbrain, frontal, medial prefrontal & occipital, cingulate cortices and ↑ atrophy in prefrontal cortex<br>SH-PD: ↓ metabolism in the regions shown at baseline and precuneus and ↑ atrophy in broader areas.
Baba et al. (2011)[2]	17 NH-PDs<br>10 MH-PDs<br>33 SH-PDs	PET	WB	Metabolism	• SH-PDs: occipital hypometabolism<br>• NH-PDs: bil. DLPFC hypometabolism<br>• Non-demented SH-PDs: medial occipital hypometabolism<br>• Odor identification was related to broad cortical dysfunction (e.g., AMG and piriform)
Bohnen et al. (2007)[3]	27 PDs<br>27 HCs	PET	ROI (dorsal striatum)	DAT binding ratio	• (+) corr between olfactory scores and DAT activities in dorsal striatum, caudate, and putamen
Bohnen et al. (2008)[4]	29 PDs	PET	ROI (HIPP, AMG, striatum)	DAT binding ratio	• (+) corr between olfactory function and DAT activities in HIPP, followed by AMG, dorsal and ventral striatum
Bohnen et al. (2010)[5]	58 PDs<br>26 HCs	PET	ROI (neocortical and HIPP areas, AMG, striatum)	AChE and VMAT2 activities	• (+) corr between UPSIT and AChE activity in HIPP, AMG, & dorsal striatum<br>• (+) corr between UPSIT and striatal VMAT2 activity<br>• AChE activity in HIPP & AMG predicted UPSIT scores
Haugen et al. (2016)[6]	183 PDs	PET	ROI (striatum)	VMAT2 activity	• High prevalence of hyposmia in PD with PET confirmed dopamine denervation

Siderowf et al. (2005)[7]	24 PDs	SPECT	ROI (striatum)	DAT binding ratio	• (+) corr between UPSIT and striatum and putamen<br>• DAT in putamen: NH-PDs > MH-PDs<br>• No difference in DAT in caudate between PD olfactory groups
Sommer et al. (2004)[8]	30 Hyposmic PDs	TCS	ROI (striatum)	Echogenicity	• 36.7% PDs with hyposmia: ↓ echogenicity of striatum
Altinayar et al. (2014)[9]	41 PDs<br>19 HCs	T2	ROI (OB)	Volume	• PD=HC
Brodoehl et al. (2012)[10]	16 PDs<br>16 HCs	T2	ROI (OB)	Volume	• HC >PD: bil. OB volume<br>• PD: OB volume (+) corr with olfactory scores
Kim et al. (2007)[11]	59 PDs<br>25 HCs	T2	ROI (OS))	Depth	• PD=HC<br>• No corr between depth of OS and olfactory scores
Ham et al. (2016)[12]	171 PDs	FLAIR	ROI (PWMH, DWMH, BG, infratentorial WMH)	WMH	• DWMH and total WMH (-) corr with olfactory function
Wang et al. (2011)[13]	29 PDs<br>29 HCs	T1 or T2	ROI (OB, OS)	OB: volume<br>OS: depth	• PD and HC: (+) corr between olfactory performance and OB volume<br>• PD and HC: No corr between olfactory performance and OS depth
Wu et al. (2011)[14]	12 Hyposmic PDs<br>14 NH-PDs<br>26 HCs	T1	ROI (olfactory-related structures)	Volume	• Hyposmic PDs vs. HCs: ↑ WM, ↓ GM in bilateral parahHIPP, Rt. OFC<br>• NH-PDs vs. HCs: ↑ WM, ↓ GM in Rt. ParaHIPP<br>• Olfactory performance (+) corr with WM, but (-) corr with GM in Rt. paraHIPP and OFC
Campabadal et al. (2017)[15]	25 PDs<br>24 HCs	T1	WB + ROI (striatum)	Cortical thickness, volume	• Baseline: HC> PD in Lt. rostral MFG & cuneus<br>• Follow-up: No difference between PD olfactory decliners and non-decliners<br>• PD: (+) corr between UPSIT change and changes in Rt. thalamus, Rt. caudate, & Lt. putamen<br>• PD olfactory decliners > non-decliners: volume loss in Rt. caudate
Chen et al. (2014)[16]	20 PDs<br>14 MSAs<br>12 HCs	3D-TSE, T1FFE	OB	Volume	• PD < HC and MSA<br>• MSA=HC<br>• PD: (-) corr between disease duration and OB volume<br>• PD: (-) corr between UPDRS-III and OB volume

Lee et al. (2014)[17]	40 PDs<br>40 HCs	T1	WB	Volume	• PD < HC: Bil. piriform, OFC, frontal pole & parietal-occipital regions<br>• PD: (+) corr between UPSIT and Rt. piriform & Lt. OFC<br>• HC: No corr between UPSIT and any brain region
Wattendorf et al. (2009)[18]	16 PDs<br>16 HCs	T1	WB	Volume	• Early PD: (+) corr between Rt. piriform and olfaction<br>• Advanced PD: (+) corr between Rt. AMG and olfaction<br>• HC: No corr between olfaction and any brain volume<br>• No difference in total brain or grey matter volume between groups
Mueller et al. (2005)[19]	11PDs<br>9 HCs	3D-CISS	ROI (OB)	Volume	• PD = HC
Moessang et al. (2011)[20]	16 PDs<br>16 HCs	Task fMRI	WB & ROI (mesolimbic & non-mesolimbic olfactory regions)	Activation	• PD> HC: activation in piriform and OFC<br>• PD: Loss of odour selectivity related to Lt. AMG, Rt. paraHIPP, bil. OFC<br>• PD: Primary olfactory cortex areas better discriminated olfaction in PD
Welge-Lüssen et al. (2009)[21]	18 PDs	Task fMRI	WB	Activation	• PDs with intact olfaction > PDs with olfactory dysfunction: AMG, paraHIPP, IFG, insula, cingulate gyrus, striatum, ITG
Westerman et al., (2008)[22]	16 HCs<br>12 PDs	Task fMRI	ROI (ACC, IFG, striatum)	Activation	• HC: Bil. AMG & HIPP<br>• PD: Lt. AMG & HIPP<br>• PD> HC: Bil. IFG & ACC, Lt. dorsal striatum. Rt. Ventral striatum
Su et al., (2015)[23]	38 Hyposmic PDs<br>16 NH-PDs<br>22 HCs	RS-fMRI	WB	ReHo, FC, volume	• ReHo:<br>Hyposmic PDs < HC: Bil. rectus, OFC, AMG, paraHIPP<br>Hyposmic PDs< NH-PDs: Bil. insula, STL; paraHIPP, OFC, IFG, OT, rectus, Rt. AMG<br>PD: (-) corr between olfactory scores and ReHo in bil. OFC, Rt. Insula<br><br>• FC:<br>Hyposmic PD < NH-PDs: limbic/ paralimbic cortices, especially in regions with reduced ReHo in hyposmic PD.
Sunwoo et al., (2015)[24]	23 PD-Hs<br>64 PD-Ms<br>23 PD-Ls	RS-fMRI	WB	FC	• PD-H, PD-M> PD-L: FC of PCC with bil. primary sensory cortex, Rt. frontal and parietal areas (1)<br>• PD-L > PD-H, PD-M: Striatocortical FC with bil. occipital and Rt. frontal areas (2)

					• Olfaction (+) corr with FC between PCC and regions in (1).<br>• Olfaction (-) corr with FC between caudate and regions in (2).
Scherfler et al., (2013)[25]	16 PDs<br>14 HCs	DTI,<br>PET,<br>TCS	ROI (SN, OT)	FA, MD,<br>echogenicity	• PD: (+) corr between DAT in putamen and olfactory performance<br>• PD> HC: MD in OT<br>• PD: MD of SN (-) corr with DAT in putamen and OT<br>• No corr between FA of SN and motor and olfactory functions<br>• PD> HC: nigral hyperechogenic area
Zhang et al. (2011)[26]	25 PDs<br>25 HCs	DTI	WB	FA, MD	• PD vs. HC: ↓ FA ↑ MD in bil. cerebellum & OFC<br>• MD: PD> HC in bilateral OFC & ITG; PD < HC in bil. parietal lobes & Lt. precentral gyrus<br>• FA: PD< HC in bil. cerebellum & Rt. Rectus<br>• PD: (+) corr between FA in Lt. cerebellum and olfactory function<br>• PD: (-) corr between MD in Rt. cerebellum and olfactory function
Ibarretxe-Bilbao et al. (2010)[27]	6 NH-PDs<br>9 SH-PDs<br>9 Anosmic PDs<br>23 HCs	DTI	ROI (OFC, primary olfactory cortex, uncinate)	FA	• Rectus:<br>Anosmic PDs, SH-PDs< HCs<br>Anosmic or hyposmic PDs< NH-PDs<br><br>• Primary olfactory cortex:<br>Anosmic PDs< HCs
Rolhelser et al., (2011)[28]	14 PDs<br>14 HCs	DTI	WB & ROI (SN, AOS)	FA, MD, RD, AD	• WB: PD=HC<br>• ROI (SN): PD < HC in FA, PD > HC in RD<br>• ROI (AOS): PD< HC in FA
Scherfler et al. (2006)[29]	20 PDs<br>20 HCs	DTI	WB	Trace	• PD >HC: Bil. OT
Georgiopoulos et al. (2017)[30]	22 PDs<br>13 HCs	DTI, MT	WB +ROI (piriform, enthorhinal cortex, OFC)	FA, MD, RD, AD, MT	• DTI (WB)<br>AD & MD: PD < HC in Lt. corticospinal tract, tracts near Lt. SN, & Rt. internal capsule.<br>AD: PD < HC in body and splenium of corpus callosum, bil. internal capsule, Lt. uncinate fasciculus & rectus<br>FA & RD: PD=HC<br>• DTI (ROI):

					MD: PD < HC in Lt. enthorhinal cortex<br>AD: PD < HC in Rt. OFC<br>• MT (WB & RO): No significant findings.
Joshi et al. (2017)[31]	24 PDs<br>26 HCs	T2, DTI	ROI (SN, AOS)	FA, MD, RD, AD	• AOS:<br>T2: PD= HC<br>FA in Rt. AOS: PD < HC<br>MD in Rt. AOS: PD > HC<br>• SN:<br>T2 & FA: PD= HC<br>MD: PD> HC
Iannilli et al. (2017)[32]	17 PDs<br>21 HCs<br>20 Hyposmic HCs<br>13 Anosmic HCs	EEG	ROI (cerebral & cerebellar cortices)	GFP, GMD	• HC> PD: Rt. ACC, paraHIPP, & angular gyrus.

Note: (+) corr: positive correlation; (-) corr: negative correlation; ACC= anterior cingulate cortex; AChE= acetylcholinesterase; AD= axial diffusivity; AMG= amygdala; AOS= anterior olfactory structures; BG= basal ganglia; bil.= bilateral; CISS= constructive interference in steady state precession; DAT= dopamine transporter; DLPFC= dorsolateral prefrontal cortex; DTI= diffusion tensor imaging; DWMH= deep white matter hyperintensities; FA= fractional anisotropy; FC= functional connectivity; FLAIR= Fluid attenuation inversion recovery; fMRI= functional magnetic resonance imaging; GFP= global field power; GMD= global map dissimilarity; HC= healthy control; HIPP= hippocampus; IFG= inferior frontal gyrus; ITG= inferior temporal gyrus; Lt.= left; MH= moderate hyposmia;; MD= mean diffusivity; MT= magnetization transfer; NH= no hyposmia; OB= olfactory bulb; OFC= orbitofrontal cortex; OS= olfactory sulcus; OT= olfactory tract; paraHIPP= parahippocampus; PCC= posterior cingulate cortex; PD= Parkinson's disease; PD-Hs= PD patients with high olfactory scores; PD-Ms= PD patients with middle olfactory scores; PD-Ls= PD patients with low olfactory scores; PET= positron emission tomography; PWMH= periventricular white matter hyperintensities; RD= radial diffusivity; ReHo= regional homogeneity; RS-fMRI= resting state functional magnetic resonance imaging; ROI= region of interest; Rt.= right; SH= severe hyposmia; SN= substantia nigra; SPECT= single photon emission-computed tomography;STL= superior temporal lobe; TCS= transcranial sonography; UPDRS-III= Unified Parkinson Disease Rating Scale-Part III;UPSIT= University of Pennsylvania Smell Identification Test; VMAT2= Vesicular monoamine transporter type II; WB= whole-brain; WMH= white matter hyperintensities.

References:

1 Baba, T. *et al.* Severe olfactory dysfunction is a prodromal symptom of dementia associated with Parkinson's disease: a 3 year longitudinal study. *Brain* **135**, 161-169, doi:10.1093/brain/awr321 (2012).

2 Baba, T. *et al.* Association of olfactory dysfunction and brain. Metabolism in Parkinson's disease. *Movement Disorders* **26**, 621-628 (2011).

3 Bohnen, N. I. *et al.* Selective hyposmia and nigrostriatal dopaminergic denervation in Parkinson's disease. *Journal of Neurology* **254**, 84-90, doi:10.1007/s00415-006-0284-y (2007).

4 Bohnen, N. I., Gedela, S., Herath, P., Constantine, G. M. & Moore, R. Y. Selective hyposmia in Parkinson disease: Association with hippocampal dopamine activity. *Neuroscience Letters* **447**, 12-16, doi:10.1016/j.neulet.2008.09.070 (2008).

5 Bohnen, N. I. *et al.* Olfactory dysfunction, central cholinergic integrity and cognitive impairment in Parkinson's disease. *Brain* **133**, 1747-1754, doi:10.1093/brain/awq079 (2010).

6 Haugen, J. *et al.* Prevalence of impaired odor identification in Parkinson disease with imaging evidence of nigrostriatal denervation. *journal of Neural Transmission (Vienna Austria 1996)* **123**, 421-424 (2016).

7 Siderowf, A. *et al.* [99mTc]TRODAT-1 SPECT imaging correlates with odor identification in early Parkinson disease. *Neurology* **64**, 1716-1720 (2005).

8 Sommer, U. *et al.* Detection of presymptomatic Parkinson's disease: combining smell tests, transcranial sonography, and SPECT. *Movement Disorders* **19**, 1196-1202 (2004).

9 Altinayar, S. *et al.* Olfactory disfunction and its relation olfactory bulb volume in Parkinson's disease. *Eur Rev Med Pharmacol Sci* **18**, 3659-3664 (2014).

10 Brodoehl, S. *et al.* Decreased olfactory bulb volume in idiopathic Parkinson's disease detected by 3.0-tesla magnetic resonance imaging. *Movement Disorders* **27**, 1019-1025 (2012).

11 Kim, J., Lee, W., Chung, E. & Dhong, H. Analysis of olfactory function and the depth of olfactory sulcus in patients with Parkinson's disease. *Movement Disorders* **22**, 1563-1566 (2007).

12 Ham, J. *et al.* Effect of olfactory impairment and white matter hyperintensities on cognition in Parkinson's disease. *Parkinsonism & Related Disorders* **24**, 95-99 (2016).

13 Wang, J. *et al.* Association of Olfactory Bulb Volume and Olfactory Sulcus Depth with Olfactory Function in Patients with Parkinson Disease. *American Journal of Neuroradiology* **32**, 677-681, doi:10.3174/ajnr.A2350 (2011).

14 Wu, X. L. *et al.* Correlation between Progressive Changes in Piriform Cortex and Olfactory Performance in Early Parkinson's Disease. *European Neurology* **66**, 98-105, doi:10.1159/000329371 (2011).

15 Campabadal, A. *et al.* Brain correlates of progressive olfactory loss in Parkinson's disease. *Parkinsonism & Related Disorders* **S1353-8020**, 1-7 (2017).

16 Chen, S. *et al.* Imaging of olfactory bulb and gray matter volumes in brain areas associated with olfactory function in patients with Parkinson's disease and multiple system atrophy. *European Journal of Radiology* **83**, 564-570 (2014).

17 Lee, E. *et al.* Olfactory-related cortical atrophy is associated with olfactory dysfunction in Parkinson's disease. *Movement Disorders* **29**, 1205-1208 (2014).
18 Wattendorf, E. *et al.* Olfactory Impairment Predicts Brain Atrophy in Parkinson's Disease. *Journal of Neuroscience* **29**, 15410-15413, doi:10.1523/Jneurosci.1909-09.2009 (2009).
19 Mueller, A. *et al.* Olfactory bulb volumes in patients with idiopathic Parkinson's disease a pilot study. *Journal of Neural Transmission (Vienna Austria 1996)* **112**, 1363-1370 (2005).
20 Moessnang, C. *et al.* Altered Activation Patterns within the Olfactory Network in Parkinson's Disease. *Cerebral Cortex* **21**, 1246-1253, doi:10.1093/cercor/bhq202 (2011).
21 Welge-Lussen, A. *et al.* Olfactory-Induced Brain Activity in Parkinson's Disease Relates to the Expression of Event-Related Potentials: A Functional Magnetic Resonance Imaging Study. *Neuroscience* **162**, 537-543, doi:10.1016/j.neuroscience.2009.04.050 (2009).
22 Westermann, B. *et al.* Functional imaging of the cerebral olfactory system in patients with Parkinson's disease. *Journal of Neurology Neurosurgery and Psychiatry* **79**, 19-24, doi:10.1136/jnnp.2006.113860 (2008).
23 Su, M. *et al.* Alterations in the limbic/paralimbic cortices of Parkinson's disease patients with hyposmia under resting-state functional MRI by regional homogeneity and functional connectivity analysis. *Parkinsonism & Related Disorders* **21**, 698-703 (2015).
24 Sunwoo, M. *et al.* Olfactory performance and resting state functional connectivity in non-demented drug naïve patients with Parkinson's disease. *Human Brain Mapping* **36**, 1716-1727 (2015).
25 Scherfler, C. *et al.* Correlation of dopaminergic terminal dysfunction and microstructural abnormalities of the basal ganglia and the olfactory tract in Parkinson's disease. *Brain* **136**, 3028-3037 (2013).
26 Zhang, K. Y. *et al.* Voxel-based analysis of diffusion tensor indices in the brain in patients with Parkinson's disease. *European Journal of Radiology* **77**, 269-273, doi:10.1016/j.ejrad.2009.07.032 (2011).
27 Ibarretxe-Bilbao, N. *et al.* Olfactory impairment in Parkinson's disease and white matter abnormalities in central olfactory areas: A voxel-based diffusion tensor imaging study. *Mov Disord* **25**, 1888-1894, doi:10.1002/mds.23208 (2010).
28 Rolheiser, T. *et al.* Diffusion tensor imaging and olfactory identification testing in early-stage Parkinson's disease. *Journal of Neurology* **258**, 1254-1260 (2011).
29 Scherfler, C. *et al.* Voxel-wise analysis of diffusion weighted imaging reveals disruption of the olfactory tract in Parkinson's disease. *Brain* **129**, 538-542 (2006).

30 Georgiopoulos, C. *et al.* Olfactory impairment in Parkinson's disease studied with diffusion tensor and magnetization transfer imaging. *Journal of Parkinson's Disease* **7**, 301-311 (2017).
31 Joshi, N. *et al.* Lateralized microstructural changes in early-stage Parkinson's disease in anterior olfactory structures, but not in substantia nigra. *Journal of Neurology* **264**, 1497-1505 (2017).
32 Iannilli, E., Stephan, L., Hummel, T., Reichmann, H. & Haehner, A. Olfactory impairment in Parkinson's disease is a consequence of central nervous system decline. *Journal of Neurology* **264**, 1236-1246 (2017).
